# Strong Expectations Cancel Locality Effects: Evidence from Hindi

**DOI:** 10.1371/journal.pone.0100986

**Published:** 2014-07-10

**Authors:** Samar Husain, Shravan Vasishth, Narayanan Srinivasan

**Affiliations:** 1 Linguistics, University of Potsdam, Potsdam, Brandenburg, Germany; 2 School of Mathematics and Statistics, University of Sheffield, Sheffield, South Yorkshire, United Kingdom; 3 Centre of Behavioural and Cognitive Sciences, University of Allahabad, Allahabad, India; University of Leicester, United Kingdom

## Abstract

Expectation-driven facilitation (Hale, 2001; Levy, 2008) and locality-driven retrieval difficulty (Gibson, 1998, 2000; Lewis & Vasishth, 2005) are widely recognized to be two critical factors in incremental sentence processing; there is accumulating evidence that both can influence processing difficulty. However, it is unclear whether and how expectations and memory interact. We first confirm a key prediction of the expectation account: a Hindi self-paced reading study shows that when an expectation for an upcoming part of speech is dashed, building a rarer structure consumes more processing time than building a less rare structure. This is a strong validation of the expectation-based account. In a second study, we show that when expectation is strong, i.e., when a particular verb is predicted, strong facilitation effects are seen when the appearance of the verb is delayed; however, when expectation is weak, i.e., when only the part of speech “verb” is predicted but a particular verb is not predicted, the facilitation disappears and a tendency towards a locality effect is seen. The interaction seen between expectation strength and distance shows that strong expectations cancel locality effects, and that weak expectations allow locality effects to emerge.

## Introduction

The role of expectations in sentence comprehension has received a great deal of attention in the psycholinguistic literature ever since Levy [1] applied the surprisal proposal of Hale [2] to account for a range of phenomena in the sentence comprehension literature (also see [3]–[5]). A central insight of the Hale and Levy expectation based account is that sharper syntactic expectations lead to faster processing time. For example, the expectation based account predicts that in a sentence like *The administrator who the nurse met…*, when the distance between the argument noun phrase *the administrator* and a verb *met* is increased by interposing another relative clause, *The administrator who the nurse that was from the clinic met…*, the expectation for a verb becomes sharper in the second case, leading to faster reading times at the verb compared to the first sentence.

One issue with this account is that it makes the wrong prediction for some cases. Expectation-based accounts do well for German [6], [7], Hindi [8] and Japanese [9], but languages like English and Russian [10] do not conform to the predictions of the expectation-based account. For example, Grodner and Gibson [11] (also see [12]) showed that increasing distance in the manner shown above results in slowdowns at the verb. This so-called locality effect has been explained in terms of the increased cost of completing a dependency when a co-dependent is more distant [13], [14]. The underlying explanation for such effects has been cast in terms of decay and interference [13], [15]–[18]: the argument is difficult to retrieve either because it has become less accessible in memory over time (decay), or because other nouns intervening between the co-dependents make it difficult to identify and retrieve the correct target noun (interference; specifically, retroactive interference). Decay and interference as explanations are difficult to disentangle; for the purposes of this paper, it is sufficient to note that these are possible alternative explanations. We will refer to the decay/interference accounts as the locality account.

Given that both expectation and locality are needed in order to explain the full range of data available, a two-factor account has been proposed [1]. Previous proposals that attempt to spell out a two-factor account have either added the two sources of difficulty into a single computational model [4], or have suggested that surprisal may show earlier effects than retrieval cost [19]. Another possibility, suggested by Levy and colleagues [10], is that expectation effects may only occur in head-final languages like German, Hindi, and Japanese, while locality effects might dominate in verb-medial languages like English and Russian. This proposal makes sense because a speaker of a head-final language might be able to retain predictions of upcoming heads better than a speaker of a non-head final language, simply because the frequency with which they see head-final structures is quite high. Vasishth and colleagues [20] have also made an argument similar to Levy and colleagues' in the context of the processing of ungrammatical center-embedded structures. They examined the observation by Gibson and Thomas [21] (due originally to Janet Fodor) that difficult-to-understand sentences like *The apartment that the maid who the service had sent over was cleaning every week was well decorated* subjectively seem easier to process if the middle verb is removed, rendering the sentence ungrammatical: *The apartment that the maid who the service had sent over was cleaning every week was well decorated*. Vasishth and colleagues carried out several eye-tracking and self-paced reading studies with English and German double center embeddings and showed that, when reading the ungrammatical sentences, English speakers experienced a facilitation immediately after the final verb was read; surprisingly, however, German speakers slowed down in the same region when the sentence was ungrammatical. The same pattern that German exhibits has been found in Dutch [22]. In their 2010 work, Vasishth and colleagues suggested that German speakers may be better able to maintain predictions of head-final structures, because they encounter them more often. They speculated that German speakers do not forget the prediction of the upcoming verbs and are therefore able to detect the ungrammaticality; English speakers might be forgetting the predictions because such structures occur only rarely. These speculations are similar to the proposal by Levy and colleagues that head-finality may be a key factor in determining whether expectation or the working-memory based locality effect dominates.

Another way that expectation and locality might together influence sentence processing difficulty is that the timing of expectation and locality effects might differ. Staub [23] showed that in English object relatives, the arrival of the subject noun phrase after the relative pronoun leads to a higher proportion of regressive saccades, compared to regressions from the object noun phrase in a subject relative. In a sentence like *The administrator who the nurse met*, at *who* a subject relative is expected and so a verb is highly expected. When the noun phrase *the nurse* appears instead of a verb, the expectation for the verb is dashed, resulting in a higher proportion of regressions. Because Staub found that reading times at the object relative verb were also higher compared to the subject relative verb (also see [11]), it is reasonable to conclude that both expectation and locality might play a role, but at different stages in parsing. There is new, independent support for the idea that these effects might co-occur but at different stages [10]. Levy and colleagues investigated reading times in Russian relative clauses, which are post-nominal. Russian (an SVO language) has relatively free word order, which allows the verb to appear clause medially or clause finally inside a relative clause. Levy and colleagues showed that when the relative clause verb was positioned further away from its arguments, a slowdown was observed, consistent with locality predictions. But they also found facilitation when a more predictable structure was built, consistent with expectation-based predictions; an accusative NP was read more slowly when it occurred in a rare position (clause initially inside the RC) than its canonical position (clause finally).

The Staub [23] and Levy et al. [10] results show that a dashed expectation results in a slowdown. However, once an expectation is dashed, does the rarity (frequency of occurrence) of the alternative structure that is built affect processing? The expectation account says yes: the rarer the alternative structure built, the greater the processing cost. We began our investigation of expectation effects by trying to establish whether this prediction is correct. We used Hindi as the target language because of its special properties, which are discussed below.

### Relative clauses in Hindi

In Hindi, the relative pronoun can be case-marked, so that it is immediately clear that a subject/object relative is being processed. Relative clauses in Hindi are often post-nominal, as in English; the postnominal RC is in no way marked. This is the relative clause type we consider in this paper. An example of subject and object relative clauses is shown in (1). Since subject relatives tend to have animate head nouns and object relatives tend to have inanimate head nouns [24], we present such prototypical examples below. In the glosses for the Hindi sentences, we use ERG to represent ergative case marking, ACC for accusative case marking, and at places mark gender with FEM/MASC, when this is relevant for expository purposes. Other markers are TOP for topics, OBL for oblique case marking, and ABS for absolutive case marking.

(1) a. vah laRkaa, jisne kitaab paDhii thii, meraa dost hai

that boy.MASC who ERG book.FEM read had.FEM my friend is

‘That boy, who read the book, is my friend.’

b. vah kitaab, jisko us laRke ne paDhaa thaa, bahut moTii hai

that book which ACC that boy read had very thick is

‘That book, which that boy read, is very thick.’

In the subject relative shown in (1a), the relative pronoun *jisne*, ‘who-ergative’, makes it clear that this is a subject relative. In (1b), *jisko*, ‘who-accusative’, signals an object relative (ergativity is explained below). The verb inside the relative clause is *paDhii thii/paDhaa thaa*, ‘read had’, and has different morphology in subject versus object relatives. This arises from the properties of the agreement system in Hindi: the verb agrees with the first argument in its subcategorization list that is not case-marked, and if all arguments are case marked (as in 1b), a default masculine third person singular morphology appears on the verb. In (1b), the object is case-marked by virtue of being co-indexed with the relative pronoun, which is case-marked.

As is clear from (1), the default word order inside the relative clause is verb-final in both subject and object relatives; this is a consequence of the fact that Hindi is a language with subject-object-verb (SOV) word order. Because Hindi is a free word order language [25]–[27], the word order inside the relative clause can be varied: the verb can be in a clause-medial or clause-final position in both subject and object relatives.

When clause-final, the verb is distant from the head noun of the relative clause, and when clause-medial, the verb is closer to the head noun. Examples are shown in (2).

(2) a. vah laRkaa, jisne paDhii thii kitaab, meraa dost hai

that boy.MASC who ERG read had.FEM book.FEM my friend is

‘That boy, who read the book, is my friend.’

b. vah kitaab, jisko paDhaa thaa us laRke ne, bahut moTii hai

that book which ACC read had that boy ERG very thick is

‘That book, which that boy read, is very thick.’

In this first experiment, we exploited two important properties of Hindi. The first is that subjects are elided quite often, but objects are elided only if subjects are elided as well. This fact about Hindi was first noticed by Prasad [28], who examined a hand-annotated corpus of Hindi for instances of elision. She found that out of 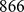 clauses, 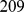 had one or more zero pronouns (an elision). Of these, 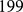 were elisions of subjects, whereas only 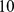 were elisions of direct objects.

A second property of Hindi that we exploited relates to ergativity in Hindi. Hindi has so-called split ergativity: when a transitive verb has perfective aspect, its subject and object respectively have ergative and absolutive case marking. The ‘split’ in split ergativity refers to the presence of ergativity conditional on verbal aspect; cross-linguistically, ergativity can occur unconditionally. Ergative case marking is expressed by the postposition *ne*, which appears on the subject, and absolutive case marking on the object; this has no overt morphology. The bare morphology of the absolutive marker is assumed in order to distinguish it from overt case-marking by the accusative marker *ko*, which we see on the relative pronoun in object relatives.

An example is shown in (3a), which is in perfective aspect; here, ergative case marking must appear on the subject and absolutive case marking can occur on the object. One could put accusative case marking on the object, (3b), but this leads to the meaning that we are talking about a specific book. Compare (3a) with (3c), where ergative case is not allowed, because the verb is not in perfective aspect.

(3) a. Raam ne kitaab paRhii

Raam ERG book ABS read

‘Raam read a book. (Ergative marking on Raam)’

b. Raam ne kitaab ko paRhaa

Raam ERG book ACC read

‘Raam read a (specific) book. (Ergative marking on Raam)’

c. Raam (*ne) kitaab paRhe-gaa

Raam (*ERG) book read-will

‘Raam will read a book. (Absolutive marking on Raam)’

Thus, when the reader encounters an ergative-case marked noun at the beginning of a sentence, a strong expectation for a transitive verb with perfective morphology is generated. In addition, unless the preceding context already mentions a specific object noun, the object of the predicted transitive verb should have absolutive marking (bare morphology).

These two facts about Hindi—the relative frequencies of subject vs object elision, and split ergativity—allow us to test a prediction of the expectation account: when an expectation is dashed, the rarity of the structure built should affect processing cost.

In subject relatives, when the relative pronoun has ergative case marking, the RC verb is essentially certain to be transitive, by the definition of ergativity. That is, an expectation is built up for a verb phrase with an object NP. Moreover, due to the verb-final nature of Hindi, the object NP is expected to appear before the verb; see (4) below for an example. The highly expected object NP is shown after the relative pronoun and is marked in bold (note that the specific lexical item is of course not predicted; only an object NP is).

(4) vah laRkaa, jisne **kitaab** paDhii thii, meraa dost hai

that boy who ERG book read had my friend is

‘That boy, who read the book, is my friend.’

In the subject relative, if a verb appears after the relative pronoun (instead of an object NP), the sentence could in principle continue without an overt object NP (i.e., one can elide the object), but such a continuation involves building a very rare structure (as discussed above, based on Prasad's work), which is odd at best:

(5) ??vah laRkaa, jisne (bahut dilchaspi se) paDhii thii, kitaab meraa dost hai

that boy who ERG (with great interest) read had book my friend is

(Lit.) ‘That boy, who read the book (with great interest), is my friend.’

Due to the difficulty in building such a rare structure, the reader should slow down when they encounter the transitive verb following the relative pronoun. This prediction, which stems from the expectation-based account, is the opposite of the prediction made by decay/interference accounts; the latter would predict a facilitation when the verb is closer to the relative pronoun. Recall that in Russian relative clauses, this prediction of the decay/interference accounts has been confirmed [10].

By contrast, in object relatives, the accusative case marking on the relative pronoun signals to the reader that a subject NP along with a transitive verb is expected. Here, the expected verb need not necessarily be in perfective aspect. This is clear from (7) below: the continuation after the relative pronoun is compatible with a perfective transitive verb (which would require *-ne* ergative case marking on the NP, see 7a) or a non-perfective transitive verb (7b).

(6) a. vah kitaab, jisko **us laRke ne** paDhaa thaa, bahut moTii hai

that book which that boy ERG read had very thick is

‘That book, which that boy read, is very thick.’

b. vah kitaab, jisko **vo laRkaa** paDhaa rahaa thaa, bahut moTii hai

that book which that boy reading was very thick is

‘That book, which that boy was reading, is very thick.’

Repositioning of the verb clause medially is rare in Hindi relatives, but possible. A search in the Hindi treebank [29] (which contains a collection of news articles from a Hindi newspaper, with 400k words and 20,969 sentences) yielded 256 subject relatives with canonical (verb-final) order, and 2 with non-canonical (verb clause-medial) order; and 87 object-relatives with canonical order, and 0 with non-canonical order. In object relatives, if the relative clause verb is re-positioned to appear before the subject NP inside the relative clause, surprise should be experienced by the reader, and a revised structure should momentarily be built: a transitive verb with an elided subject NP. An elision of the subject NP is likely because, as Prasad has shown, eliding the subject is quite common in Hindi. This revised structure should not be as difficult to build as in the subject relative, because subject elision is quite frequent compared to object elision. In the elided subject construction shown below, the elided noun refers by default to the speaker:

(7) vah kitaab, jisko bahut dilchaspi se paDhaa thaa, bahut moTii hai

that book which with much interest read had very thick is

‘That book, which (I) read with great interest, is very thick.’

In order to find independent evidence for this asymmetry of object vs subject elision, we also carried out a sentence completion study. Participants were asked to complete the non-canonical (verb clause-medial) version of subject and object relative clause as shown in (8). The items used in the sentence completion study corresponded to the non-canonical conditions of the experimental items discussed in the next section. 24 sets of items, each with three versions were presented using the centered self-paced reading method in the standard Latin square design. Unlike the non-cumulative self-paced moving window method, in the centered self-paced reading method each region appears in the center of the presentation screen. Items were presented using Douglas Rohde's Linger software, version 2.94 (http://tedlab.mit.edu/~dr/Linger/). Out of the three conditions presented during the study, (8) shows the two conditions relevant for experiment 1, the third condition (not shown here) was unrelated to the study discussed here. The front-slashes in (8) indicate the partitioning of the sentence presented during the study. The critical items were presented with 122 filler items unrelated to this study. Twenty-one subjects participated for payment. Their mean age was 22.7 years, SD 3.1 years.

(8) a. Subject relative

vah laRkaa, /jisne/bahut dilchaspii se/paDhii thii/…

that boy who ERG with much interest read had…

b. Object relative

vah kitaab, /jisko/bahut dilchaspii se/paDhaa thaa/…

that book which ACC with much interest read had…

The sentence completion study confirms Prasad's [28] findings. Out of 168 instances, participants produced an overt subject in object relatives such as (8b) in only 4 instances. Compared to this, they produced an explicit object NP in subject relatives such as (8a) in 100 instances. This shows that Hindi native speakers find it easier to elide a subject compared to a direct object.

It follows from the above discussion that, according to the expectation-based account, repositioning the RC verb clause-medially in subject relatives should cause greater difficulty (due to having to build a VP with an elided object) compared to a similar repositioning of the RC verb in object relatives (where the reader must build a VP with an elided subject).

An alternative outcome, predicted by the decay/interference accounts, is a slowdown whenever the verb is more distant from the head noun. In other words, the decay/interference accounts of Gibson [13] and Lewis and Vasishth [14] uniformly predict faster reading times whenever the relative clause verb occurs verb medially. We tested these predictions in Experiment 1.

## Experiment 1

### Participants

Sixty native speakers of Hindi in Allahabad, India, participated for payment. Their mean age was 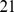 years, SD 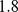 years.

### Design

Example items are presented below. We used a 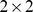 design: Relative Clause Type (Subjects vs Object Relative), and Verb Distance (Distant vs Near). An adverbial or prepositional phrase which modified the relative clause verb was placed just before the relative clause verb in order to ensure that the region before the RC verb was always the same. The front-slashes mark the grouping of the phrases for presentation to participants (discussed below).

(9) a. Subject relative, Distant (Canonical order)

vah laRkaa, /jisne/kitaab/bahut dilchaspii se/paDhii thii, /meraa dost/hai

that boy who ERG book with much interest read had my friend is

‘That boy, who read the book with great interest, is my friend.’

b. Subject relative, Near (Non-canonical order)

vah laRkaa, /jisne/bahut dilchaspii se/paDhii thii/kitaab, /meraa dost/hai

that boy who ERG with much interest read had book my friend is

‘That boy, who read the book with great interest, is my friend.’

c. Object relative, Distant (Canonical order)

vah kitaab, /jisko/us laRke ne/bahut dilchaspii se/paDhaa thaa, /

that book which ACC that boy with much interest read had

bahut moTii/hai

very thick is

‘That book, which that boy read with great interest, is very thick.’

d. Object relative, Near (Non-canonical order)

vah kitaab, /jisko/bahut dilchaspii se/paDhaa thaa/us laRke ne, /

that book which ACC with much interest read had that boy

bahut moTii/hai

very thick is

‘That book, which that boy read with great interest, is very thick.’


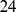
 sets of items were constructed, each with the four versions shown above. These were arranged in the standard Latin square design, which ensures that each participant sees 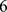 unique versions of each condition in a counterbalanced manner. In addition to the target items, 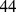 fillers were pseudo-randomly interspersed. These sentences had a variety of constructions: simple declarative clauses, different kinds of ellipses, clausal complements, relative clauses, etc. Half the target sentences (12 items) and 11 of the fillers were followed by comprehension questions which were about different dependencies in the sentence to prevent participants from developing a strategy for answering the questions. Because it is so expensive to travel to India to conduct experiments, and because of the restricted time available to run experiments while in India, in addition to the target items and the fillers mentioned above, items from three other experiments were interspersed. One of these experiments was experiment 2. All items, data, and R and Stan code used in this paper are available from either of the first two authors.

### Procedure

We used the non-cumulative self-paced moving window method [30]. Stimulus items were presented using Douglas Rohde's Linger software, version 2.94 (http://tedlab.mit.edu/~dr/Linger/). The Latin square design ensured that each participant saw each item in only one condition. The target items and fillers were pseudo-randomized for each participant.

The experimenter began by explaining the task to the participants. After this, several practice sentences were presented in order to familiarize participants with the task. At the beginning of each trial, the computer screen showed a row of hyphens that covered the upcoming sentence; when the space bar was pressed, the first word or phrase was unmasked. The grouping of the phrases is shown in (9). With each successive press of the space bar, the next word or phrase was unmasked, and the previously unmasked segment was masked again. This successive unmasking continued until the participant had read the whole sentence. Reading times or RTs (in milliseconds) were taken as a measure of relative momentary processing difficulty. The f-key was pressed for answering a question with a ‘yes’ response and the j-key was pressed for answering with a ‘no’ response.

### Predictions

It may be helpful to review the predictions with reference to the specific target items before looking at the results. The region of interest in all the conditions is the relative clause verb *paDhii thii* (subject relatives), or *paDhaa thaa*, (object relatives), ‘had read’. As mentioned earlier, the difference in verb morphology arises from the agreement system in Hindi.

Within each relative clause type, the decay/interference accounts predict uniformly slower reading times in the verb-final structures (9a,c) compared to the verb-medial structures (9b,d).

The predictions of the expectation-based account are different by relative clause type. In subject relatives, due to the ergative case marking on the relative pronoun, an object NP and a transitive verb with perfective morphology are predicted; since the canonical order is Object Verb, the object is expected to appear before the verb. In (9a), after the relative pronoun, the object and verb appear, as expected; this is the canonical ordering. The purpose of the adverb, which always precedes the verb, is to keep the pre-critical region constant in the distant and near conditions. In contrast to (9a), in (9b) the relative pronoun is followed by the adverb and the verb. This is predicted by the expectation account to lead to a slowdown because it is a rare construction.

In object relatives, the relative pronoun is accusative case marked, which leads to a prediction of a transitive verb with no particular aspectual morphology, and a subject NP, with word order Subject Verb. In (9c) no difficulty should be experience upon encountering a subject NP, adverb and verb. But in (9d) the relative pronoun is followed by the adverb and the verb; when the verb is encountered, a revised prediction is made of a transitive verb with an elided subject. Therefore, here too, we expect a slowdown. In both relative clause types, when the expectation is dashed, an alternative structure is momentarily built, with an elided noun phrase. Building this alternative structure is harder in subject relatives than object relatives because it is more difficult to elide an object than a subject, as discussed above. This means that the slowdown due to the early appearance of the verb in the subject relative is expected to be larger than the slowdown in the object relative. Thus, we expect an interaction between the factors relative clause type and distance: a greater slowdown in subject relatives than object relatives due to the verb occurring clause medially.

In summary, the decay/interference accounts predict longer reading times whenever the relative clause verb is more distant; by contrast, the expectation account predicts shorter reading times whenever the relative clause verb is more distant, and an interaction between the factor distance and relative clause type.

### Results

#### A note on the statistical analyses

Sum contrasts (±1) were used to code main effects and interactions. Although question-response accuracies are not of central importance for testing the predictions of the alternative theories of interest, we include analyses of these for completeness. Binomial responses in the question-responses were analyzed using generalized linear mixed models with a logit link function (using the **lme4** function in R); subjects and items were crossed random factors. Maximal models were fit, in the sense of Barr and colleagues [31], when this was feasible: a full variance-covariance matrix was fit for both subjects and items, with variance components for intercepts and slopes. If there was a convergence failure or the correlation estimates were 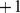 or 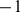, models were fit without the correlation estimation, and if the model still did not converge, a reduced variance structure for items was used (intercepts only).

The models for reading time, which was the main dependent measure of interest, were fit using Stan [32] and the R package **rstan** (Version 2.0.1, packaged 25th October, 2013), using uninformative priors for all variance components and for the fixed effects (see Materials and Methods for details). We fit these models using Stan rather than **lme4** for several reasons. First, with the arrival of Stan, it has for the first time become possible to fit complex hierarchical models in a Bayesian framework. Second, we wanted to fit maximal models in order to make the most conservative inference possible given the structure of the data [31]. Such maximal models did not converge in **lme4**, but are possible to fit (with convergence) in Stan, by using appropriate weakly informative priors for the variance components [33]. A third reason was that Bayesian models allow us to directly test the weight of the evidence in favor of a specific hypothesis to be tested. For example, if one theory predicts that a particular parameter 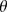 is less than 0 (or greater than zero), we can directly test the evidence for or against these theories given the data by examining the posterior probability of the parameter being less than or greater than 0. This is a more direct evaluation of the evidence for the research hypothesis at hand than computing conditional probabilities like p-values, which only allow us to reject a null hypothesis like 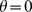, without telling us anything directly about the research hypothesis (a low p-value doesn't tell us anything about the *specific* hypothesis being tested, it can only lead us to reject the null).

Reading time was transformed to the log scale. Hypothesis testing for the reading time data was done by examining the posterior probability of the parameter being positive or negative given the data. We calculated the posterior probability by sampling the relevant parameter 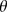 from the posterior distribution (see Materials and Methods for details), and computing the probability that 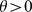 or 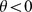 (depending on whether the estimate had a positive or negative sign, respectively).

#### Question-response accuracies and question-response times

The accuracies for the four conditions were: subject relatives, verb distant: 91%, subject relatives, verb near: 88%, object relatives, verb distant: 86%, object relatives, verb near: 82%. Object relatives had lower accuracy, and the distant configurations had higher accuracy as shown in Table 1 below. The distance 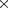 RC type interaction was marginal, as was the effect of distance within subject relatives and within object relatives. We found no effect in question-response times.

**Table 1:** Experiment 1: The main effect of distance, of RC Type, and their interaction on question-response accuracy; and the effect of distance within subject and object relatives.

ANOVA contrasts			
*comparison*	*coef.*	*SE*	*z-score*
Distance	0.51	0.18	2.83
RC Type	−0.47	0.22	−2.16
Distance 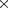 RC Type	−0.25	0.15	−1.70

Nested contrasts			
*comparison*	*coef.*	*SE*	*z-score*
Distance [SR]	0.34	0.20	1.69
Distance [OR]	0.29	0.16	1.80

### Reading time analyses at the critical region

The reading times at the critical region were the main dependent measure of interest; Table 2 presents the relevant analyses. A main effect of distance was found, with slower reading times when the verb was closer to the relative pronoun, as predicted by expectation accounts. A main effect of relative clause was found, such that object relatives were read more easily than subject relatives; and evidence for an interaction between distance and relative clause type was found, such that the slowdown in subject relatives due to re-positioning the verb medially was much larger than the corresponding slowdown in object relatives. A nested contrast showed that the larger effect of distance in the subject relative (estimated coefficient 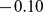; cf. the estimated coefficient for object relatives, 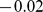) was responsible for the overall longer reading times in subject relatives compared to object relatives.

**Table 2:** Experiment 1: The main effect of distance, of RC Type, and their interaction on reading times at the critical region; and nested contrasts showing the effect of distance in SRs and ORs.

ANOVA Contrasts			
*comparison*	*coef.*	*95% credible interval*	*Posterior prob.*
Distance	−0.06	[−0.09, −0.03]	0.99
RC Type	−0.05	[−0.08, −0.02]	0.99
Dist 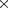 RC Typ	0.04	[0.01, 0.07]	0.99

Nested Contrasts			
*comparison*	*coef.*	*95% credible interval*	*Posterior prob.*
Distance [SR]	−0.10	[−0.14 −0.06]	0.99
Distance [OR]	−0.02	[−0.05 0.02]	0.82

The table shows the estimated coefficients, the 95% credible intervals, and the posterior probability of the parameter being positive (or negative, depending on its sign), given the data. This model was fit using a Bayesian maximal linear mixed model using Stan.

**Figure 1:**
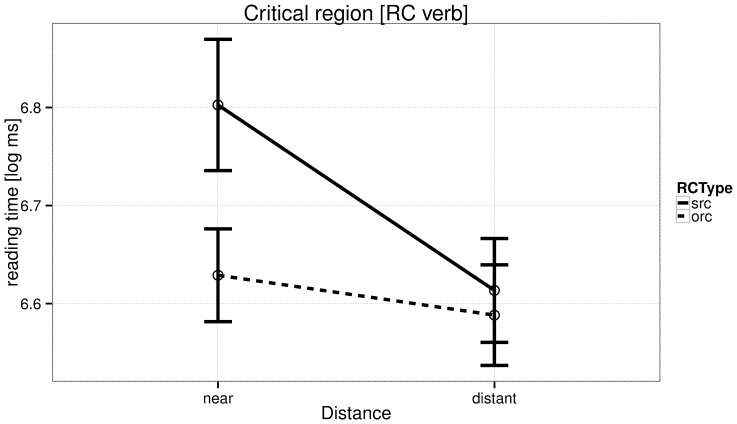
Experiment 1: Reading times in log ms at the critical region (relative clause verb) for the four conditions.

**Figure 2:**
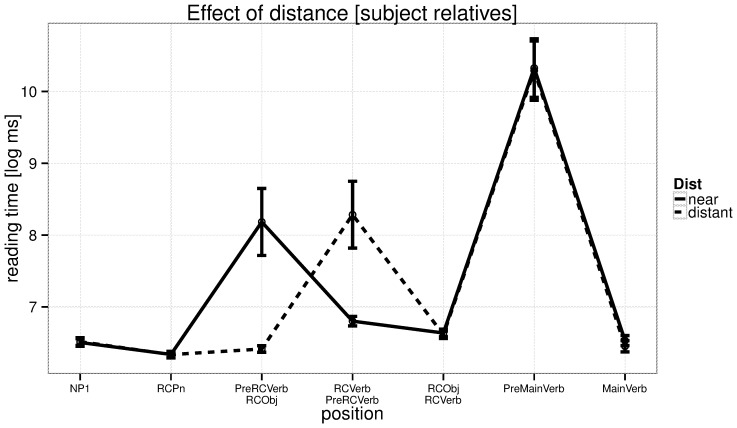
Experiment 1: reading times by region in subject relatives. The critical region is RCVerb.

**Figure 3:**
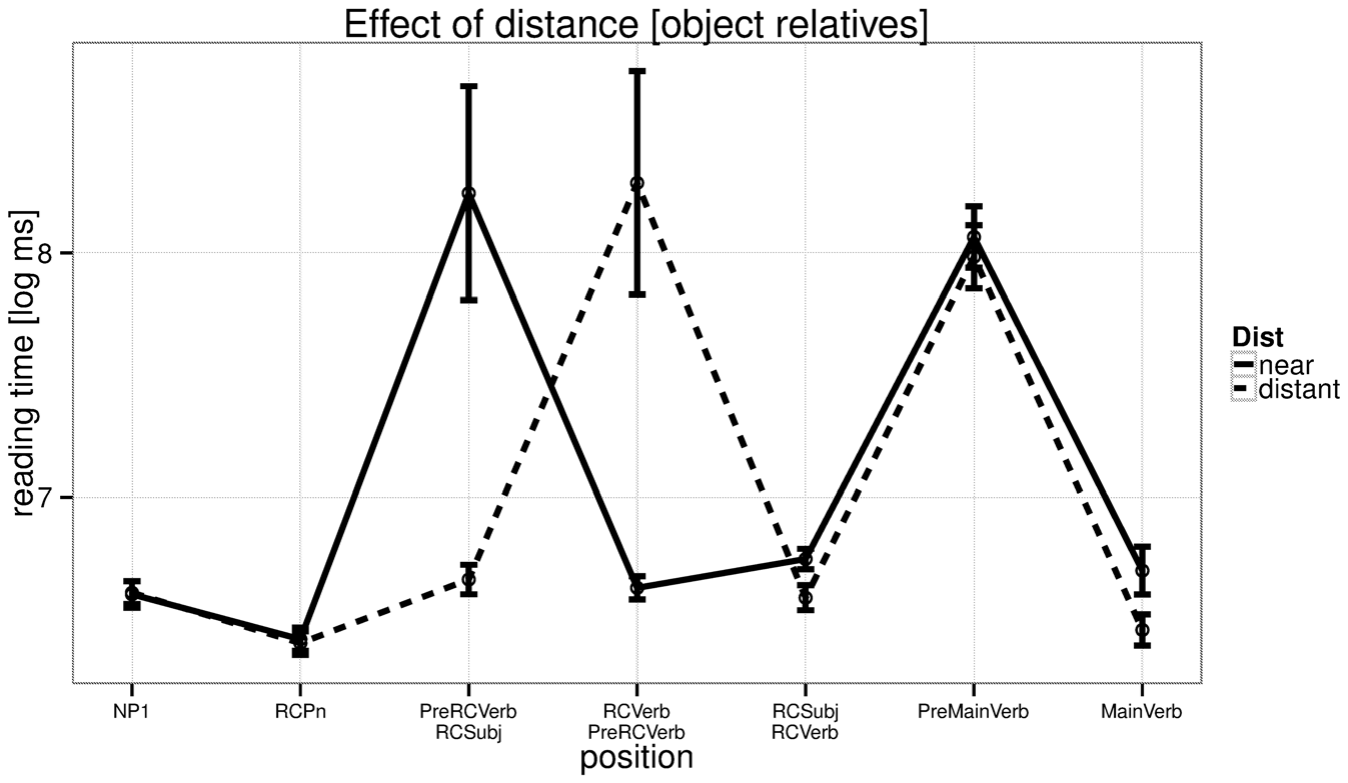
Experiment 1: reading times by region in object relatives. The critical region is RCVerb.

The reading times at the critical verb are shown in Figure 1, and reading times for all regions are shown for subject relatives in Figure 2 and for object relatives in Figure 3. We discuss the patterns in the by-region plots in the discussion section.

### Discussion

Experiment 1 shows that a dashed expectation is costly: in subject relatives, we see a slowdown when the relative clause verb is closer to the relative pronoun. The faster reading time at the RC verb in the distant condition as compared to the near condition is not a consequence of facilitation due to more intervening material between the relative pronoun and the RC verb; rather this difference arises due to the disruption in processing caused as a result of non-canonical (verb clause-medial) order in the ‘near’ condition. This is consistent with previous findings by Staub [23] and Levy and colleagues [10]. The distance 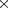 RC Type interaction seen in Experiment 1 also confirms a further prediction of the expectation account: revising a dashed prediction to a rarer structure, the object NP elision in subject relatives, is more costly than revising a dashed prediction to a less rare structure, the elided subject in object relatives.

The results can be explained by the pre-building of syntactic structure of as yet unseen words based on predictions triggered by the previous context (in this case, the relative pronoun). The ergative case-marker on the relative pronoun can predict the nature of the upcoming verbal head in a language like Hindi. More importantly, because this verb has been predicted, the argument structure and the word order information associated with the verb become accessible to the parser. These constraints are then employed to drive the expectation of the parser. Specifically, because of split-ergativity in Hindi, the ergative case on the relative pronoun in conditions 9(a) and 9(b) predicts an object NP and a transitive verb with perfective aspect. This prediction of a preverbal object is met in 9(a), while in 9(b) it is not met, causing a slowdown due to a dashed expectation.

In the object relative, the accusative case on the relative pronoun in conditions 9(c) and 9(d) predicts a subject NP and a transitive verb, but this prediction for the verb is underspecified as regards aspectual marking. This prediction of a subject NP is met in 9(c), while in 9(d) it is not, causing slowdown in 9(d) due to a dashed expectation. As explained earlier, in Hindi, because it is easier to drop a subject compared to an object, the distance 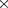 RC type interaction effect can be explained by assuming the parser's sensitivity to this asymmetry.

One concern with these results could be that the effect we see at the verb may be due to differential amounts of spillover from the pre-critical regions. From previous work [8] we know that, in an experiment design such as the present one, reading time from the preceding word can influence the reading time at the current word. It was to avoid having different words preceding the critical verb that we always had an adverb preceding the verb; this minimizes the chances that differential amounts of spillover would influence the result. Although one cannot rule out that spillover plays a role from two words back, note that such a spillover effect would predict the opposite pattern to that found in Figures 2 and 3. Specifically, in the near conditions, we should see faster reading times at the verb because two words before it is a short relative pronoun; in the distant conditions, the verb is preceded two words back by a noun phrase. If spillover were to occur from two words back onto the verb, the effect should be larger from a noun phrase (distant conditions) than from a relative pronoun (near conditions). In fact, we find a longer reading time at the verb in the near conditions.

Another possible explanation for the speed-up seen in the distant conditions is the word-position explanation: there could be a tendency towards a general increase in reading speed as one progresses through a sentence (see [34] for a discussion about this issue). It could be argued that in the distant conditions, the speed-up which we attribute to expectation effects may be due simply to the fact that we are comparing reading times at verbs in different positions. There are at least two reasons why this explanation does not make sense. First, Levy and colleagues [10] carried out a series of self-paced reading studies that had the same design as the present experiment, and they found slow-downs at the verb in the more distant conditions. If there were a *general* tendency for a speed-up with increasing word position, we would not see such a pattern. A second reason why the word-position effect cannot explain our results is the interaction we find between word order and relative clause type. The word-position explanation would incorrectly predict a similar magnitude of effect (no interaction) in both relative clause types.

As an alternative to the expectation-based account outlined above, it is worth considering a simpler frequency-based account where the parser is sensitive only to the patterns of different phrase ordering in the language. If such a pattern-frequency-based explanation can account for the results then it would be a much simpler explanation compared to prediction driven by case marking. In order to test this possibility, we calculated the relevant statistics on relative clauses using the Hindi treebank [29], which contains a collection of news articles from a Hindi newspaper, with 400k words and 20,969 sentences. Table 3 shows that encountering a relative clause verb immediately after a ‘relative pronoun-erg, adjunct’ sequence is rare (a ‘relative pronoun-erg, adjunct, RC verb’ sequence corresponds to the SRC-Near condition). Similarly, encountering an RC verb in ORC-Near condition is also a rare occurrence. A similar pattern emerges when one draws these phrase order frequency counts from monoclausal sentences instead of relative clause sentences. For example, a verb following an ‘NP1-erg adjunct’ or an ‘NP1-acc adjunct’ sequence is a rare occurrence. The rare occurrence of the verb in medial position in RCs can easily explain the slowdown seen in these two conditions. However, as can be verified from Table 3, such a frequency-based account cannot explain the shape of the interaction effect found in the result. Thus, our explanation, in terms of the relative rarity of the transition made after an expectation is dashed, accounts for the observed patterns better than a parsing system that relies only on frequency counts of phrase order.

**Table 3:** Treebank-based frequency for phrase order in conditions b, d in Experiment 1.

Condition	Region	Count	
		*RC Verb*	*Other*
SRC-Near	rel.pron-erg adjunct	1	9
ORC-Near	rel.pron-acc adjunct	1	20

‘adjunct’ signifies a time/place/manner adverbial, ‘Other’ signifies any other phrase that is not relative clause verb.

The results therefore point to a parsing mechanism where a syntactic expectation results in a configuration with longer dependency length (9a,c) as opposed to a shorter one (9b,d). The experiment shows that the well-known preference for local dependencies over non-local ones can be canceled by a language-specific predictive parsing system. In the present case, a prediction of a particular argument structure with a word order of ‘Subject Object Verb’, is still the preferred configuration in spite of an increased distance between subject and verb compared to a more local configuration of ‘Subject Verb…’. As noted earlier, in the present experiment the constraints that drive the expectation of the comprehension system arise from the high predictability of a particular type of verb (here, a transitive verb).

The results of this experiment clearly go against the predictions of the decay/interference accounts of Gibson [13] and Lewis and Vasishth [14]. Note that it is difficult to simply reject decay/interference as a factor because of the wide range of empirical phenomena that it can explain that expectation cannot [10]–[13], [15], [16], [18], [35]. Why would we see such a strong expectation effect that it cancels out locality? We hypothesized that perhaps the reason that expectation-based effects are so strong in experiment 1 is that a very specific prediction is made in subject relatives when the relative pronoun is read: a verb with a particular valency (transitive) is predicted. We speculated that the expectation effect may dominate when such a strong prediction for a particular verb type is made. A corollary of this hypothesis is that locality effects may emerge if the prediction is just for a verb without much of a commitment about the verb's properties. We investigated this possibility in experiment 2.

## Experiment 2

Experiment 1 demonstrated the effects of case-marker driven prediction. In experiment 2, we investigated whether the strength of the expectation can lead expectation effects to dominate. This was achieved by manipulating the strengths of expectation contingent on the left syntactic context.

In order to systematically manipulate expectation strength, we made use of an interesting phenomenon in Hindi, the Noun-Verb complex predicate (CP) [36], [37]. In Hindi (and Urdu), Object-Verb sequences are either (i) complex predicates, that is, a nominal predicate (also called a nominal host) followed by a highly predictable verb (a so-called ‘light verb’) which leads to a non-compositional meaning (e.g., *khayaal rakhnaa*, ‘care keep/put’; ‘to take care of’), or (ii) simple predicates, that is, object-verb sequences with a strictly compositional meaning (e.g., *gitaar rakhnaa*, ‘guitar keep/put’; ‘to put down or keep a guitar’). In both cases, the verb is lexically the same, but the meaning of the noun-verb sequence differs.

As discussed in the literature on Hindi/Urdu complex predicates [36], [37], Object-Verb complex predicates are similar to simple predicates in that they are composed in the syntax; they are not stored in the lexicon as unitary lexical entries. Mohanan [38] provides evidence that shows that the nominal host and light verb in an Noun-Verb complex predicate together form a phrase and that they are composed post-lexically, not lexically. Further evidence comes from the fact that, in Hindi, a nominal host acts like a regular argument of a verb. For example, a light verb agrees with its nominal host and a nominal host can be passivized (for more details see, [38], [39]).

This fact allows us to use these two classes of noun-verb sequences, complex predicates vs simple predicates, to systematically manipulate expectation strength. In order to manipulate distance, we added adverbials between the nominal predicate/object and its verbal head. Two to three adverbials modify the verb in ‘long’ conditions, while ‘short’ conditions had one to two adverbials. For example in (10) the complex predicate *khayaal rakhnaa* has two adverbials intervening, *binaa kisi laaparvaahi ke* ‘without any carelessness’ and *achCe se* ‘properly’.

(10) maa ne bachche ko skUla CoRaa Ora usse kahaa ki vah apnaa **khayaal** mother ERG child ACC school dropped and to her said that she her care binaa kisi laaparvaahi ke achCe se **rakhe**, phir vah apne daftar ki ora chal paRii without any carelessness properly keep, then she towards her office proceeded ‘The mother dropped the child off at the school and asked her to take care of herself properly without any carelessness, she then proceeded towards her office.’

### Pre-test to select the appropriate noun-verb sequences

In order to verify whether the verb (eg. *rakhe*, ‘keep’, in 10) in a complex predicate is strongly predicted at the noun (eg. *khayaal*, ‘care’, in 10), we carried out a sentence completion study to select an appropriate set of noun-verb sequences. We extracted a list of complex predicates from the Hindi treebank [29] and used these as a basis for the sentence completion task. In the complex predicate condition, a sentence that was a candidate target item in our experiment was presented up to the nominal host; this can be seen in example in 11(a) where *khayaal* ‘care’ is the last word of the incomplete sentence. Similarly, in the simple predicate condition, the last word of the incomplete sentence is *gitaar* ‘guitar’ which can in principle be the object of an upcoming verb.

(11) a. maa ne bachche ko skUla CoRaa Ora usse kahaa ki vah apnaa khayaal …

mother ERG child ACC school dropped and to her said that she her care

b. maa ne bachche ko skUla CoRaa Ora usse kahaa ki vah apnaa gitaar …

mother ERG child ACC school dropped and to her said that she her guitar

The study was done in two stages. In the first stage, two lists each containing 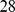 incomplete sentences were prepared (these were analogous to the sentence fragments in 11 above); these were pseudo-randomly interspersed with 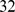 filler sentences, which were also incomplete sentences. A list contained an item either in the complex predicate condition or the corresponding simple predicate condition. Ten native-speaker participants were assigned to each list. Participants were asked to complete the sentence. The dependent measure was the proportion of cases where the correct verb was provided by the participants. This test allowed us to identify highly predictable complex predicates and simple predicates that were not highly predictable.

In this first stage, we identified 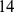 items that were suitable as experimental items. Since we had estimated that we needed at least 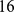 items for the experiment, in a second stage, we repeated this sentence completion task with a new set of 7 participants, with 5 target items and no fillers. This allowed us to find 2 more suitable sets of sentences. Thus, using this sentence completion test, we were able to identify configurations where the verb was highly predicable in the complex predicate case but not in the simple predicate case.

The sentence completion study confirmed that the nominal predicate in the complex predicate setting (11a) strongly predicts a light verb, while object nouns in the simple predicate setting (11b) don't predict any particular verb: The mean probability of predicting the verb in the complex predicates conditions was 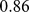, and in the simple predicate conditions this probability was 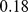.

One concern with the above completion study could be that the items were presented only up till the nominal host/object and did not contain the precritical region (i.e., the intervening phrases between the nominal host/object and the verb, cf. 10, 13a–d). It could be argued that such a study does not reflect the predictions made for the actual experimental items which include the precritical region. In order to address this concern, we conducted another sentence completion study using experimental items of experiment 2 (cf. 13a–d). 16 sets of items, each with four versions were presented using the centered self-paced reading method in the standard Latin square design. Items were presented using Douglas Rohde's Linger software, version 2.94. Example (12) shows the long conditions, the other two conditions were similar to (13b,d). The front-slashes in (12) indicate the partitioning of the sentence presented during the study. The critical items were presented with 130 filler items unrelated to this study. Twenty-one subjects participated for payment; these were the same participants who did the sentence completion study in Experiment 1. Their mean age was 22.7 years, SD 3.1 years.

(12) a. Complex predicate (long)

maa ne/bachche ko/skUla/CoRaa/Ora/usse/kahaa/ki/vah/apnaa

mother ERG child ACC school dropped and to her said that she her

/khayaal/binaa kisi laaparvaahi ke/achCe se/…

care without any carelessness properly…

b. Simple predicate argument-verb (long)

maa ne/bachche ko/skUla/CoRaa/Ora/usse/kahaa/ki/vah/apnaa

mother ERG child ACC school dropped and to her said that she her

/gitaar/binaa kisi laaparvaahi ke/achCe se/…

guitar without any carelessness properly…

Table 4 shows that the second study successfully replicated the first completion study. It shows that in the complex predicate conditions the exact light verb is predicted in 74.98% instances out of the total number of instances, while in the simple predicate conditions, the percentage prediction for the exact verb is just 17.85%. The study shows that in the simple predicate condition, there is a marginal effect of distance (t(15) = −1.96, p-value  = 0.06; mean of differences −0.75, 95% CI −1.56, 0.06).

**Table 4:** Sentence completion study results for all conditions in Experiment 2.

Condition	Total instance	Exact prediction	Percentage
Complex Predicate, long	84	60	71.40%
Complex Predicate, short	84	66	78.57%
Simple Predicate, long	84	9	10.71%
Simple Predicate, short	84	21	25.00%

In addition to controlling the prediction strengths, the frequencies of nominal hosts and objects were also controlled so as to be comparable across items. These counts were derived from a treebank corpus. The mean for the condition (a) noun was 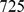 (SE 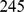) and for the condition (c) noun 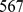 (SE 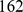). The mean difference in frequencies of the two nouns were not significantly different (t(15)  = 0.90, p-value  = 0.4; mean of differences 158, 95% CI −221, 537).

All frequency counts in experiment 2 were extracted by combining multiple corpora (Total word count  = 26,477,020): (1) EMILLE corpus (written and parallel data): 12,769,720 words; (2) ERDC parallel corpus: 223,368 words; the ERDC corpus was prepared by Electronic Research and Development Centre, NOIDA, India, it has text in different domains; (3) Hindi wikipedia text: 13,157,578 words; The material available at http://dumps.wikimedia.org/backup-index.html was parsed using: WikiExtractor (version 2.1), http://medialab.di.unipi.it/wiki/Wikipedia_Extractor; (4) The Hindi treebank (pre-release version): 326,354 words.

### Participants

Sixty participants in Allahabad, India, took part in this experiment. These were the same sixty participants that took part in Experiment 1.

### Procedure

The procedure was the same as in Experiment 1.

### Design

This was a 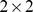 design, with Predicate Type (Complex vs Simple) and Distance (Long vs Short) as factors. In (13a), the distance between the noun and the complex predicate verb is increased by interposing adverbs: *binaa kisi laaparvaahi ke*, ‘without any carelessness’, and *achCe se*, ‘properly’. The same adverbs were interposed between the noun and the simple predicate in (13c). The conditions (13b,d) served as the baseline conditions for the complex and simple predicate cases respectively.

(13) a. Complex predicate, Long

maa ne/bachche ko/skUla/CoRaa/Ora/usse/kahaa/ki/vah/apnaa

mother ERG child ACC school dropped and to her said that she her

/khayaal/binaa kisi laaparvaahi ke/achCe se/rakhe, /phir/vah/apne

care without any carelessness properly keep, then she her

/daftar ki ora/chal paRii

towards office proceeded

‘The mother dropped the child off at the school and asked her to take care of

herself properly without any carelessness, she then proceeded towards her

office.’

b. Complex predicate, Short

maa ne/bachche ko/skUla/CoRaa/Ora/usse/kahaa/ki/vah/apnaa

mother ERG child ACC school dropped and to her said that she her

/khayaal/achCe se/rakhe, /phir/vah/apne/daftar ki ora/chal paRii

care properly keep, then she her towards office proceeded

‘The mother dropped the child off at the school and asked her to take care of

herself properly, she then proceeded towards her office.’

c. Simple predicate argument-verb, Long

maa ne/bachche ko/skUla/CoRaa/Ora/usse/kahaa/ki/vah/apnaa

mother ERG child ACC school dropped and to her said that she her

/gitaar/binaa kisi laaparvaahi ke/achCe se/rakhe, /phir/vah/apne

guitar without any carelessness properly keep, then she her

/daftar ki ora/chal paRii

towards office proceeded

‘The mother dropped the child off at the school and asked her to keep her guitar

properly without any carelessness, she then proceeded towards her office.’

d. Simple predicate argument-verb, Short

maa ne/bachche ko/skUla/CoRaa/Ora/usse/kahaa/ki/vah/apnaa

mother ERG child ACC school dropped and to her said that she her

/gitaar/achCe se/rakhe, /phir/vah/apne/daftar ki ora/chal paRii

guitar properly keep, then she her towards office proceeded

‘The mother dropped the child off at the school and asked her to keep her guitar

properly, she then proceeded towards her office.’

The front-slashes indicate the partitioning of the regions of interest in the self-paced reading study. The same fillers as in Experiment 1 were present in this study.

### Predictions

Expectation-based accounts would predict a main effect of distance, i.e., processing the verb (regardless of whether it is in the complex predicate or simple predicate condition) should be easier in the more distant condition than the near condition. If, as we hypothesized, strength of expectation affects the degree of facilitation, we would also predict an interaction between prediction strength and distance; speed-ups due to distance in the complex predicate (CP) condition should be more pronounced than the simple predicate (SP) condition. This is because, while in the CP condition both the precise lexical item and the location of the verb are expected, in the SP condition only the location of the main verb is expected.

By contrast, decay/interference accounts would predict uniformly longer reading times at the verb in the long conditions, i.e., a main effect of distance. More interestingly, however, if both locality and expectation are active simultaneously, then this would predict a speed-up at the verb in the long condition of the CP condition, but a slowdown in the long condition of the SP condition. Such an interaction would imply that a strong expectation can cause locality effects to disappear, and a weak expectation can cause locality effects to appear.

### Results

#### Question-response accuracies and question-response times

The mean question-response accuracy for the complex predicate condition in the long distance condition was 77%, and for the corresponding short condition had accuracy 87%. In the simple predicate case, the accuracy for the long-distance case and the short-distance case were comparable, at 78% and 77%, respectively. We found no significant differences in any comparisons in accuracies, and no significant differences in question-response times.

#### Reading time analyses at the critical region

**Figure 4:**
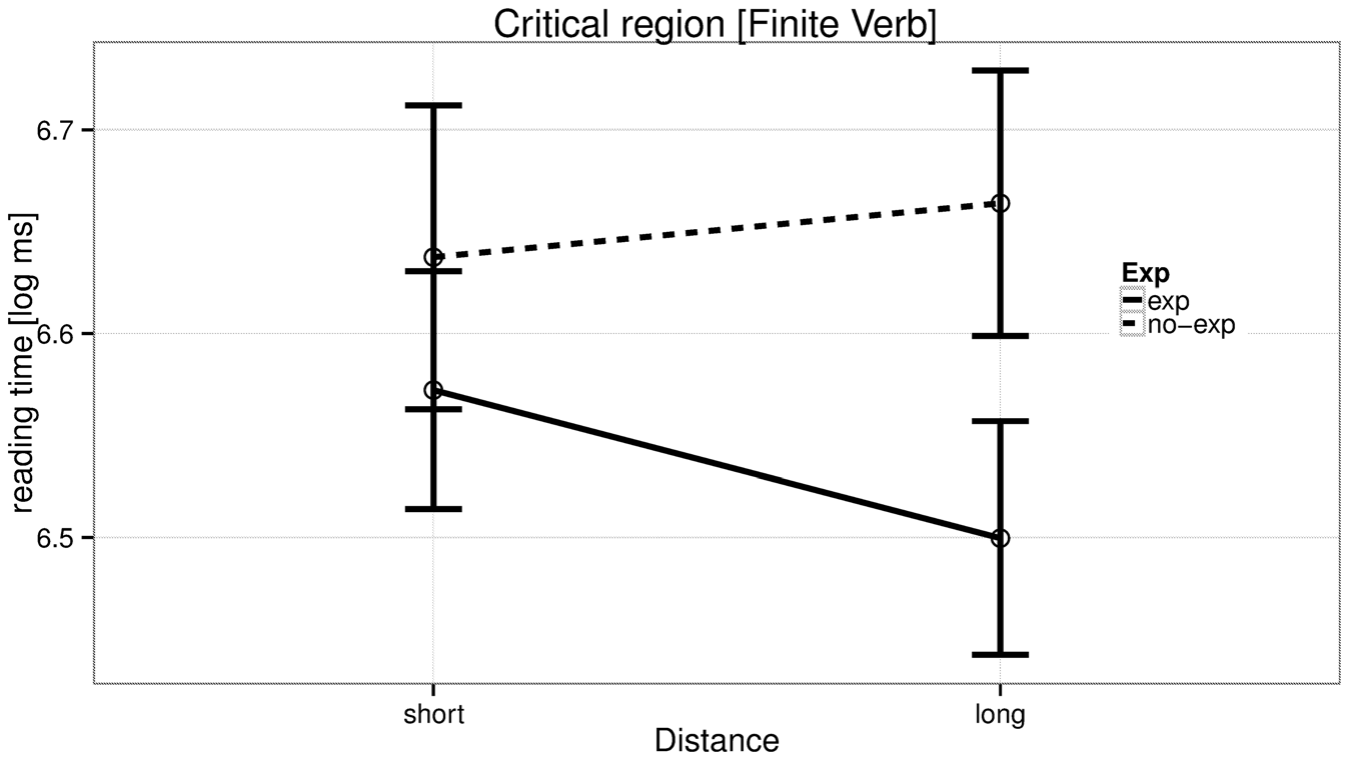
Experiment 2: Reading times in log ms at the critical region (light/simple verb) for the four conditions.

**Figure 5:**
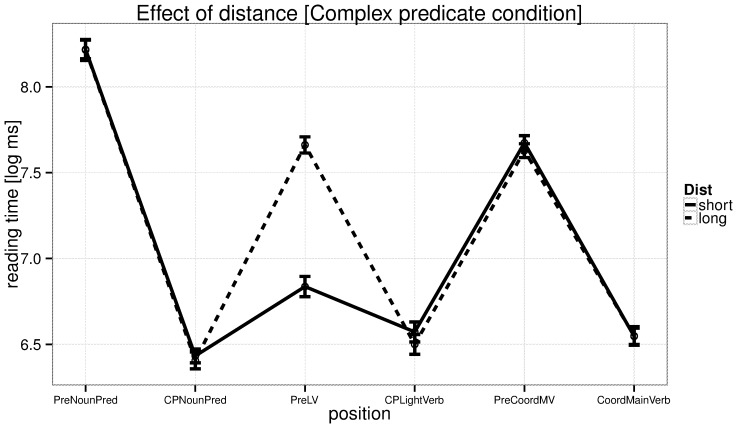
Experiment 2: reading times by region in the complex predicate condition. The critical region is CPLightVerb.

**Figure 6:**
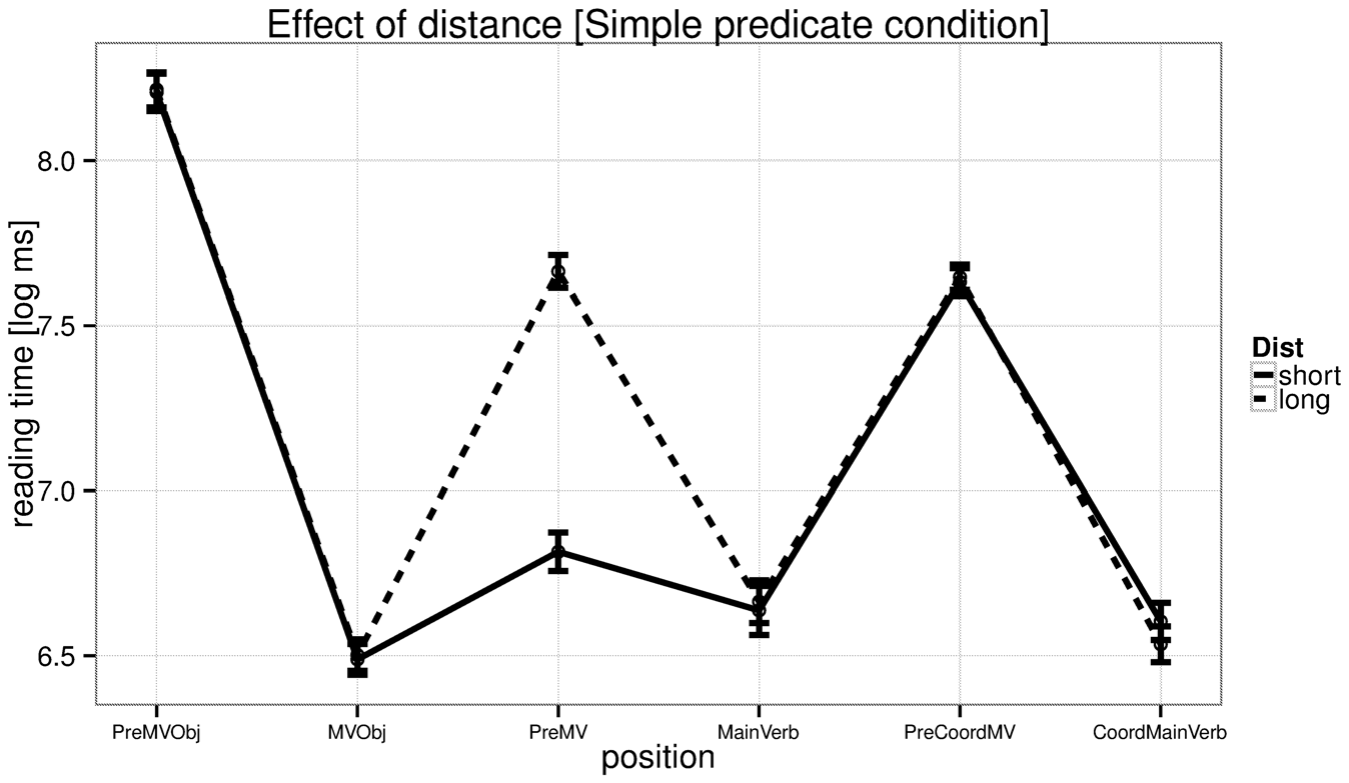
Experiment 2: reading times by region in the simple predicate condition. The critical region is MainVerb.

The reading time analyses at the critical region (the verb) are summarized in Table 5, and the effects of distance and predictability, and their interaction, are shown in Figure 4. Figures 5 and 6 provide by-region plots for the complex predicate and simple predicate conditions, respectively.

**Table 5:** Experiment 2: The main effect of distance, of predictability, and their interaction on reading times at the critical region; and effects of distance within the complex predicate and simple predicate conditions.

ANOVA contrasts			
*comparison*	*coef.*	*95% credible interval*	*Posterior prob.*
Distance	0.01	[−0.04, 0.07]	0.66
Expectation	−0.06	[−0.11, −0.01]	0.97
Dist 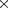 Exp	0.03	[−0.02, 0.07]	0.77

Nested contrasts			
*comparison*	*coef.*	*95% credible interval*	*Posterior prob.*
Distance [Comp. Pred.]	0.04	[−0.04, 0.13]	0.80
Distance [Simple Pred.]	−0.01	[−0.09,0.08]	0.60

The table shows the estimated coefficients, the 95% credible intervals computed using a Bayesian maximal linear mixed model, and the posterior probability of the parameter being positive (or negative, depending on its sign), given the data.

The table shows evidence in favor of expectation, such that when the specific verb was highly expected, reading time was faster; and not very compelling evidence for distance, that is, there is only weak evidence that increasing distance causes a slowdown in reading time. There is evidence for an interaction (posterior probability 0.77), in the direction predicted by our strength of expectation proposal: the distance manipulation in the complex predicate case leads to a stronger facilitation than in the simple predicate case. A nested contrast coding (effect of distance within complex predicates and within simple predicates) shows a facilitation due to increased distance in complex predicates, and a tendency towards a locality effect in the simple predicate condition. The post-critical regions showed no effects.

### Discussion

To summarize, we found strong evidence for expectation at the critical verb such that the high expectation (complex predicate conditions) were read faster than the low expectation (simple predicate) conditions; not much evidence for an effect of distance, that is, there was no evidence that increasing distance causes a slowdown; and substantial evidence for an interaction, such that the difference in reading time between long and short conditions in complex predicates was larger and in the opposite direction than the difference between long and short conditions in simple predicates.

The main effect of expectation was driven by the complex predicate. The complex predicate verb that later appears in the experimental sentence is highly expected after the nominal host has been read; our sentence completion study established that. This facilitation due to high expectation reduces the effect of memory retrieval cost at the verb. One interesting possibility is that, because the specific verb is so predictable in a complex predicate, the verb could already be integrated into the predicted VP when the nominal host is processed. If the predicted VP already contains the verb, the dependency between the nominal host and the verb could be completed at the nominal host itself. Such a mechanism would explain the absence of a locality effect in the complex predicate case. Immediate completion of a dependency at the nominal host may be one way to characterize what it means to have a strong expectation.

In the case of the simple predicate, we observe the opposite pattern; here we see a tendency towards a slowdown when distance is increased. Recall that in the simple predicate condition the probability of predicting the exact verb at the noun is very low (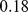). As a result, the dependency between the noun and the verb can only be completed when the main verb is eventually read. That would mean that the object (and other dependents) are retrieved and integrated when the verb is first encountered. Such retrievals and dependency building processes are known to be resource intensive [13], [14]. One implication of the contrast between complex and simple predicates, which needs to be tested in future work, is that no interference effects should be observed in the complex predicate (since no retrieval is needed at the verb), compared to the simple predicate.

The crucial piece of evidence in this experiment for our strength-of-expectation hypothesis is the interaction between distance and expectation. This interaction shows that, in the complex predicate condition, increasing distance results in a speedup, but no such speedup is seen in the simple predicate condition. The speedup in the complex predicate case can be understood as increased expectation of seeing the (already predicted, exact) verb due pruning of alternative structures [1].

Note that our account of interaction of locality and expectation is different from that of both Vasishth and Drenhaus [19] and Levy and Keller [40]. Both these works propose that locality and expectation interact such that the latter is dominant only when working memory load is relatively low, i.e., one would expect to see expectation effects in simple structures and locality in complex constructions. Our theoretical contribution here is to identify another factor that can influence the interaction between locality and expectation effects, namely, prediction strength. Under this view, prediction strength is defined as being strong if the lexical item/class-type of the predicted item is predictable with a high degree of certainty, and prediction strength is defined as weak if the part of speech verb is predicted, but the exact identity of the verb is not predictable. In the present experiment, this was true for the complex predicate condition where the light verb was strongly predicted at the nominal host; and in the simple predicate condition, the verb was weakly predicted at the object noun. These data show that, holding the complexity of the syntactic structure (and therefore memory load) constant, if prediction strength is strong, we see facilitation, and if prediction strength is weak, the locality effect begins to manifests itself. This data cannot be fully explained either by expectation-based theories [1], [2], or by decay/interference accounts [13], [14].

## General Discussion

In experiment 1 we found evidence from Hindi relative clauses that revising a dashed expectation to a rarer structure is more costly than revising a dashed prediction to a more frequent structure. In experiment 2 we found that, given left context, when a *particular* verb is predicted with near certainty, delaying its appearance results in a facilitation; but, in the same syntactic configuration, when a verb is predicted, also with near certainty, but the verb's exact identity is not predictable, we see a tendency towards a slowdown. An interesting observation that is worth pointing out relates to the sentence completion study in experiment 2 (Table 4). The study shows a marginal effect of distance in the simple predicate condition such that there are fewer exact predictions in the long condition compared to short; this trend also shows up in the complex predicate condition. Such a pattern suggests that, during sentence completion (as opposed to sentence comprehension), production planning might be adversely affecting prediction maintenance leading to verbal predictions getting decayed with increased distance. This forgetting effect needs to be further investigated.

The first experiment confirms an important prediction of the expectation-based account [1], [2] that the processing cost will be higher for a rarer transition compared to a less rare transition. Interestingly, the Hindi results are quite different from the recent findings by Levy and colleagues [10] on Russian relative clauses, which had the same design as our experiment 1. The Russian studies found resounding evidence in favor of decay/interference accounts: distant (clause-final) verbs were read more slowly. How can we reconcile these two results? One possibility is that in Hindi, the expectation for a clause-final positioning of the verb is so strong that dashing that expectation leads expectation effects to dominate. A clear implication of this proposal is that it should be possible to “switch off” or at least reduce the impact of expectation effects if the expectation is “weak” in some sense. In experiment 2, we operationalize weak and strong expectations by comparing simple and complex predicate constructions; in our experimental items the verb in the complex predicate construction is highly predictable given the left context, while in the simple predicate case the particular verb is not predictable, but the fact that some verb will appear is predictable. Here, strong expectation amounts to predicting an exact lexical item, and weak expectation amounts to predicting only an upcoming verb (identity unknown). In the former situation, speedups consistent with the expectation account are seen, while in the latter situation, we see a tendency towards a slowdown. Crucially, we see an interaction between expectation type and distance. This suggests that when expectations are strong, they may overwhelm locality effects; and when expectations are weak, locality effects start to become visible. An interesting aspect of this result is that we find an attenuation of an expectation effect in a head-final language; until now, head-final languages like German, Japanese, and Hindi have only shown expectation effects. Our work suggests that head-finality may not be a crucial factor in determining whether expectation effects dominate; but expectation-strength may be.

## Materials and Methods

### Ethics statement

The Ethics Commission of the University of Allahabad, India has provided written approval of this project. Subjects did not have to sign an informed consent form because the data were collected in an anonymized manner.

### Fitting Bayesian linear mixed models using Stan

Here, we provide a brief description of how the linear mixed models were fit using Stan [32], which is a programming language developed for fitting Bayesian models.

The general setup for the analyses of reading time was as follows. Assuming a 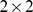, design, i.e., two factors A and B, each with two levels coded 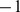 and 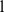; 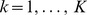 items; and 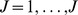 participants, log reading time 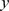 can be written down as a function of the following predictors:
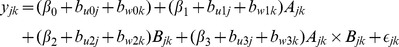
(1)where the adjustment to the various 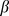, the varying intercepts and slopes by subject and item (random effects), are assumed to be distributed as follows:
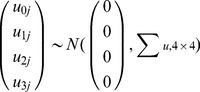
(2)and
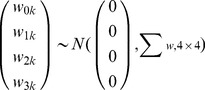
(3)



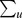
 and 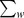 are the variance-covariance matrices for the random effects. In the Stan models, we define uninformative (uniform) priors to the 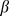, and define an uninformative prior on the variance-covariance matrices by specifying an uninformative or mildly informative priors on the respective correlation matrix [33]. The prior is a random correlation matrix specified by a single parameter, 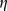, which can be considered to be the shape parameter of a symmetric beta distribution [41]. If 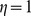 then its distribution is jointly uniform; if it's greater than 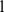, then the distribution is concentrated around the identity matrix, which has 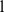
's in the diagonals and 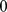
's in the off-diagonals (recall that it's a multivariate distribution), and as 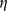 increases, the distribution becomes more sharply concentrated around the identity matrix. If 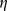 lies between 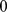 and 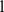, then there is a trough at the identity matrix. More details are provided in the Stan manual [42]. In the present models, we used 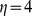, although the results do not depend on this choice.

For each model, four chains were run for 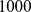 iterations each, after a warm-up (or burn-in) of 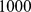. Convergence was checked using visual inspection of the chains and by computing the Gelman-Rubin convergence diagnostic [43]. The code, data, and stimulus items (including fillers) associated with this paper are available from either of the first two authors.
